# Indigenous Sundanese Leadership: Eco-Systemic Lessons on Zero Emissions: A conversation with Indigenous leaders in Ciptagelar, West Java

**DOI:** 10.1007/s11213-022-09606-y

**Published:** 2022-08-24

**Authors:** Ida Widianingsih, Janet Judy McIntyre, Ugi Sugriana Rakasiwi, Gustaff Harriman Iskandar, Rudolf Wirawan

**Affiliations:** 1 Center for Decentralization and Participatory Development Research, Faculty of Social and Political Sciences, Universitas Padjadjaran, Bandung, Indonesia; 2 University of South Africa, Pretoria, South Africa; 3 Adelaide University, Adelaide, Australia; 4 Kasepuhan Ciptagelar Community, Cisolok, Indonesia; 5 Universitas Padjadjaran and Common Room Neworks, Bandung, Indonesia

**Keywords:** West Java, Indigenous knowledge systems, Systemic balance with nature, Re-generation

## Abstract

‘Ecology of mind’ (Bateson, 1972) is a key concept applied to this project engaged in by a social anthropologist, a policy researcher, and a Sundanese Chief. Together we explore how the agendas for COP 26 could be attained and to what extent the Nobel economist Elinor Ostrom’s (2018) eight principles are relevant for managing the commons and key learnings that can be shared more broadly.The paper details the Sundanese forest community’s organisational systems to support living in ways that re-generate and sustain the forest and the way that the community has connected with Universitas Padjadjaran and an NGO called the Common Room Networks (common room.id), in order to support and extend their learning with nature program within and beyond Indonesia. Decolonising and learning from Indigenous leadership can be fostered through forming communities of practice between universities and indigenous leaders. The paper discusses how the Ciptagelar community demonstrates low carbon living and how they have organised agriculture in terms of a seasonal calendar. It makes the case that sharing their agricultural methods and community lifestyle to lower emissions could help to inspire others to follow their re-generative approach to governance and their organisational strategies. The paper demonstrates the relevance of Ostrom’s principles which are considered in relation to the case study. On the basis of a series of conversations held via zoom and email we make a case for learning from the leadership rooted in the Sundanese culture and demonstrated in West Java First Nations. It can be read as a twin paper with the paper on the Venda forest, titled ‘Eco-centric living: a way forward towards zero carbon’.

## Introduction and Statement of the Problem

In Indonesia landlessness and deforestation is a cause of poverty and places food security at risk. The Ciptagelar Village provides a case study of Indigenous wisdom that exemplifies systemic praxis by protecting forests and biodiversity. We discuss the notion of ecocentric approaches and draw parallels with Bien Vivier and the plea made by Chilisa (2018) for learning from indigenous knowledge systems and avoiding the presumption that modernisation approaches will enhance life chances. Poverty, climate change and pandemics suggest that learning from indigenous communities that have sustained their lifestyles for generations is now overdue (McIntyre-Mills 2021a, b, c, d, e, f).

The aim is to discuss the Indigenous systemic approach to forest protection and to draw out lessons from the Ciptagelar Indigenous villagers on how to achieve zero emissions through protecting the forest, through the way they organise their relationships with one another and nature. The paper will focus on agriculture and food security, infrastructure, energy, education and governance and how their way of life demonstrates zero carbon living. As the AVAAZ summary stresses a great deal of organising occurred outside and within the formal meetings to try to raise the issue of deforestation and the importance of finding alternatives to a carbon based, fossil fuel economy.

The Nobel peace prize nominee for 2020 is Chief Raoni who made the point that the world needs to stand together to protect forests.

The rationale for working on case studies across Australia, Indonesia and South Africa is that all three have in common a history of colonisation and all three face the challenges of high rates of urbanisation and associated threats to biodiversity and all three have indigenous wisdom communities which provide praxis insights on zero carbon living. This paper builds on a community of practice spanning case studies in the three regions that have been documented in a series of sole and joint authored volumes over the past three decades by the second author.

## Praxis and Key Concepts

In this paper we discuss Elinor Ostrom’s 8 principles^1^ for managing the commons, namely:

1. “Define clear group boundaries.
2. Match rules governing use of common goods to local needs and conditions.
3. Ensure that those affected by the rules can participate in modifying the rules.
4. Make sure the rule-making rights of community members are respected by outside authorities.
5. Develop a system, carried out by community members, for monitoring members’ behavior.
6. Use graduated sanctions for rule violators.
7. Provide accessible, low-cost means for dispute resolution.
8. Build responsibility for governing the common resource in nested tiers from the lowest level up to the entire interconnected system.”

This paper is twinned with another joint paper with indigenous leaders in South Africa titled: “Ecocentric living: a way forward towards zero carbon. It is structured as a conversation about Indigenous law and leadership based on custodianship and praxis in which we discuss in terms of Elinor Ostrom’s 8 principles^2^ and in which we stress (McIntyre-Mills et al., forthcoming, 2022) that:

The commons is a key concept studied by Elinor Ostrom leading to a Nobel prize in economics in 2009 (see Ostrom 2008, 2010, 2018). She focused on how indigenous communities around the world manage water sources, fisheries, forests, grazing meadows and developed 8 principles that will be explored in this paper, but like Shiva (2012, 2020a, b)  and Higgins et al. (2013) we argue the need for a global law to protect the commons and all the inhabitants of a region and to prevent its destruction by individuals and companies. We need local management to address the following issues explored by Ostrom^3^:

“Who has right to access the commons? What are their user rights? How are the commons monitored? Who decides the rules? How much can people withdraw ***(from commonly held lands and waters)***? What are the sanctions for those who break the rules? How is conflict resolved?”^4^

Ostrom stresses a) the need to think of implications for the whole and create positive externalities and not pass on the problems for future generations. b) the need to work together and to stop the barriers to interdisciplinary work at universities and c) the importance of local participation and bottom up approaches,^5^ and her empirical research demonstrates that Hardin’s (1968) argument that *shared or common areas will inevitably be managed badly is not inevitable* and most importantly, the argument is unsubstantiated.^6^ Ostrom also stresses that Hardin’s  thesis is not based on substantiated research and that Llyod’s (1833 thesis) has been used as a justification for privatisation which has not protected the commons! This point is also made by Shiva (2002) who also makes the point that despite the need to promote local management practices, these need to be buttressed by forms of international law that can protect the commons, because no matter how hard and well people work at the local level, the commons are under threat unless they are secured *through global laws*.”

The ability of communities such as Ciptagelar to maintain food security during droughts (Iskandar 2018, 2021; Humaeni et al. 2018) is one of the many reasons why the example set by this forest community provides lessons as to how to achieve re-generative living based on protecting the environment whilst establishing high agricultural yields. Studying this community’s successful nurturing of natural resources provided (Kusdiwanggo 2016a, 2016b; Pratiwi and Kusdiwanggo 2018) inspiration for this paper as did the serving of the delicious rice by Gustaff when I was in Bandung. Universitas Padjadjaran (Unpad) and The Common Room Networks (based in Bandung, West Java) as well as the long standing networks established by the first author whose mentor (Prof. Kusnaka Adimihardja) was known to the community. The sister of the chief also studied Sundanese Language in Indonesian Education University (Universitas Pendidikan Indonesia) in Bandung. In many ways the ongoing relationship between Unpad and the West Javanese remote communities and academic researchers has helped to develop a community of practice (Wenger 1998; Wenger et al. 2002, 2009) which has enabled participatory action research using mixed methods (McIntyre-Mills 2019) to support transformation (Mertens 2017, 2019; Widianingsih and Mertens 2019) and sharing ideas. Our engagement is through email, WhatsApp, Zoom and through face to face engagement prior to the Covid-19 pandemic. The case study employs mixed methods for transformation (Mertens 2019) relying on Zoom engagement^7^ during the Covid 19 pandemic, email conversations plus face-to-face engagement by the lead author. The role of Unpad in collaboration with the Common Room Networks has helped to support the dissemination of this agricultural knowledge that has stood the test of time and that has been based on empirical testing over the millennia. It uses a participatory action research approach together with the community. The paper is written in the form of a meta dialogue in line with an eco-systemic approach which we define for the purposes of this paper as living with nature and appreciating the process of co-determination of people and nature. The community value nature and regard it as ‘sacred’ and thus the commodification of rice – around which they build their life- is considered profane (Douglas 1978). This notion of the sanctity of maintaining a balance between people, nature and the agricultural process underpins the governance and education process in Ciptagelar.

This paper is based on an interview with Chief Abah Ugi (Ugi Sugriana Rakasiwi), and Gustaff Iskandar, through a convened metalogue led by Janet McIntyre with first author Widianingsih and her colleague Gustaff Iskandar who has conducted research with Chief Abah Ugi for several years (Iskandar 2018, 2021). The engagement builds on a long standing community of praxis as a result of the first and second author working together during her higher degree studies at Flinders University and Widianingsih’s ongoing community research as executive director of her Center for Decentralization and Participatory Development Research at Universitas Padjadjaran. The first author contributes data based on her fieldwork in 2019, prior to the onset of the pandemic. The second author has acted as a mentor and visiting research fellow and together they have collaborated on research projects and hosted a Mixed Methods Symposium in 2018 with the support and contributions from Donna Mertens on transformative research (McIntyre-Mills et al. 2019).^8^

Praxis is the process of applying theory in practice to exploring re-generative living. This paper explores the way in which Indigenous agriculture has been applied for over 600 years in West Java (Tarina et al. 2021), through an in-depth conversation we explore the forest knowledge and how it could support regenerative living in other parts of Indonesia and elsewhere. Their experience shows that sustainable living does not go far enough, we need to ensure that we share a way of life that protects and nurtures organic seeds and ensures that agriculture is not developed at the expense of nature, but instead it is developed in harmony with the forest. The empirical learning has been passed down for generations as re-generative living based on a relational approach to multiple species and an appreciation that human beings are interbeings whose survival is intertwined with other living systems. As Wahl (2016, 2019) sums up sustainability is not enough, we need to learn how to live in ways that re-generate.

An ‘ecology of mind’ (Bateson 1972) is the notion that we need to think in terms of our relationships with other species, not only in terms of categories as detailed elsewhere.^9^ The idea of extending a sense of solidarity to others is explored more deeply in this paper. Whilst the second author draws on her experience of working with Indigenous leaders in Africa, the first author draws on her personal engagement in participatory action research in the village as part of her role as Executive Director of Center for Decentralization and Participatory Development Research at Universitas Padjadjaran and her role as Vice Dean for Learning, Students and Research Affairs at the Faculty of Social and Political Sciences Universitas Padjadjaran to foster engagement and transformative research. Solón (2018) defines the notion of Ben Vivien or Vivir Bien as

“to learn to live together in this complex interplay of being”. Solón continues in the same reference by explaining:

“The concept of Vivir Bien (or Buen Vivir) gained international attention in the late twentieth century as people searched for alternatives to the rampage of neoliberalism. Imperfect translations of the Andean concepts of suma qamaña and sumaq kawsay, Vivir Bien and Buen Vivir reflect an indigenous cosmovision that emphasizes living in harmony with nature and one another. As these ideas’ popularity has grown, however, their meaning has been compromised. Governments in Bolivia and Ecuador incorporated Vivir Bien and Buen Vivir, respectively, into their constitutions and governing agendas on paper, but not in spirit. Rather than radical alternatives to the dominant paradigm of development and progress, these concepts have become new branding for (un)sustainable development. The lessons are clear: to avoid state co-optation, truly revolutionary change must be based on emancipation and self-determination from below.”

## Metalogue

Janet: Is the village isolated only by geography or do you also have to apply to the Chief and councillors before entering the village? So is there a definite boundary which needs to be negotiated prior to entry? From your narrative it seems as if entry is restricted to invited guests.

Ida: The Ciptagelar community speak Sundanese, but  they also understand Indonesian language, except those elderly who are over 70 years old. The traditional elders said that the recent Kasepuhan Ciptagelar community is different as their descendants have more contact with the outside world. He said that they are no longer hiding like their ancestors; they are currently adapting the modern life style and they blend with other communities whilst holding their own cultural roots. Furthermore, the Ciptagelar community also respects the Indonesian government and obeys their rules and regulations and solves their own problems through working together and through self-help. In their words “nyanghulu ka hukum, nunjang ka nagara, mufakat jeung balarea”, which means that they “ bow to the law and they support the country.”

However, once I was also told that only those who have good intentions will be able to reach the village. Another elder also told me that most possibly I was able to travel there because of my destiny and my own roots.

The Kasepuhan Ciptagelar community was founded as a result of the nomadic life of their ancestors and oral history states that the community started to practice the nomadic tradition in 1368 from their first settlement in Kasepuhan Cipatat Urug; their latest migration was in 2000 to Kasepuhan Ciptagelar. The community strives to maintain the core values of working with nature rooted in the history of Indonesia’s Sundanese culture rooted in Hindu and Buddhist values. The Ciptagelar Indigenous village is part of a larger Sundanese forest community. The chief and elders live in an area known as Kampung Gede where the traditional government is located. No exact statistics are available on how many people identify themselves as Kasepuhan Ciptagelar community, but in 2018 the chief told us that the community comprises approximately 25,000 to 30,000 people (Iskandar 2018). Only the residents living in 100 hamlets are still practicing strong traditions. The term ‘Ngalalakon’ refers to religious rituals,^10^ involving physical and cultural spaces which provide the basis structure for their settlements which are linked with a nomadic cycle dictated by the dreams of the chief.^11^ The moving process is called *hijrah wangsit –* the calling from the ancestors (*karuhun),* the Chief will receive a vision *(Uga)* that will direct him to oversee the future. The nomadic cycle cannot be determined by humans, it normally comes through dreams, semedi or special rituals. Every time they move, they will carry the magical rice barn (*leuit* si Jimat), *pangkemitan* (the praying place)*, pangnyayuran* (cooking utilities) and *ajeng wayang golek* (puppet shows set).^12^

**Janet:** Please tell us about the way the commons are managed?

**Ida:** As an agrarian community, they have two differing rice field characters –the huma and sawah (dry and wet rice fields). Both of the rice field types embrace strong cultural history (Astutik et al. 2018, Kusdiwanggo 2014, 2017). The ancestors of Kasepuhan Ciptagelar community will open new settlement and land. The old residentials will be cared for by their children and grandchildren. This is part of expanding the territories (Kusdiwanggo and Pamungkas 2017). The king has prime ministers and so-called Rorokan (village administrators) who cover the management of a range of areas ranging from food and rice to water management. For example , three years ago they appointed a minister in charge of micro hydroelectric power. The chief said he had no interest in traditions until his father passed on and he felt called to follow in his father’s footsteps. He said that he believes that the mystical energy of the forest entered him and he was able to take on the role of chief. The chief was born in 1985 and lives in the largest house in the village built in 1938, but the villagers can trace their history over 300 years as farmers managing the forest. They are led by a chief and a Prime Minister, but the ancient system of governance is continuing to evolve. They live sustainably. The Chief studied electronics and his 18 year old daughter is studying education in Bandung. A young man who won a scholarship to Japan to study agriculture continues to farm in the traditional manner. The wisdom of the elders is respected and passed on through an oral culture supported by the community’s own radio and TV station.

The customary institutional system was developed mainly on the basis of family genealogy and the residents identify themselves as a big family. The chief is called ‘Abah’ as he is the father of the indigenous community who ensures the sustained practice of all daily activities that are bounded by strong culture and traditions. His wife is called ‘Emak’ or mother of the community, as she plays important roles in the system. Abah works together with his wife (emak). The chief is also supported by village elders known as the Rendang Kande and village government officials called Rorokan. The Rorokan have distinctive responsibilities. For example, Rorokan Padukunan is responsible to lead all traditional rituals, whilst Rorokan Pamakayaan is responsible for all agricultural activities (Iskandar 2018). Two government systems are applied in Kasepuhan Ciptagelar and adhered to by indigenous people, namely the indigenous government system and the formal government system (Hapsari et al. 2019) which connects with the wider world. Traditionally, Abah is also assisted by the Rendang Kande (personal assistant) and a number of the Rorokan (village officials) who have specific roles and responsibilities. The traditional Village government (Rorokan) is appointed based on their bloodline, regardless of gender. The traditional system also recognises the importance of democratic structure. Democratic engagement is supported by hamlet representatives which consist of village elders (Kolot Lembur or Sesepuh Lembur) who give advice to Abah in managing and taking strategic decisions in the community. Furthermore, a team of younger generation participants play a role as the technical support team that are called the Baris Rendangan. The members of the Baris Rendangan are chosen from the family of village elders, they work under the coordination of the Rorokan (care takers). The rorokan are in turn supported by a team called barisan. The appointment of the Rorokan are selected based on a traditional bloodline, spiritual guidelines, and their level of competence. All of the Rorokan are empowered to discuss important issues with Abah Ugi.

The members of the Rorokan have different functions and responsibilities, the tradition recognises different task forces, namely: The Rorokan Jero is responsible for strategic internal affairs. This is similar to the function of ministry of home affairs in modern government institution. The Rukokan Kadukunan is responsible for traditional ritual activities and belief system. The Rorokan Kapenghuluan are religious caretakers, whilst the Rorokan Pamakayaan are responsible for managing and coordinating all activities related to agricultural activities, in particular to certain rituals in the paddy cultivation process. Water resources are managed by the Rorokan Manintin or Ulu-Ulu under the coordination of Rorokan Pamakayaan who are the village officials responsible for agricultural activities.

Those who manage the water resources have three main functions: protecting the rice field, managing daily water consumption and the micro hydro. Ngarawunan is a ritual of paddy protection in dry paddy field and wet paddy field from physical, which is held by the ranks of rorokan pamakayaan (Kasepuhan wealth deposit holders) (Kusdiwanggo & Budiharta, 2020). The Rorokan Paninggaran is the food security caretaker and pamageran is the ritual of protecting the dry paddy field from disturbance. These roles are carried out by rorokan paninggaran (security deposit holders and hunters) (Kusdiwanggo & Budiharta, 2020). The Rorokan Bengkong is the circumcision caretaker and the Rorokan Pantun is the caretaker for art and performance.

The customary institution can also develop new roles which are adapted to certain needs and situations. For example, when the people of Kasepuhan Ciptagelar started to utilize the micro-hydro power plant back in 1997, a new position was created called ‘Juru Turbin’ (microhydro technician and operator) who works under the responsibility of Rorokan Jero. The micro hydro turbin is managed by the Rorokan Turbin, who works under supervision of Rorokan Pakakas/Rorokan Pandai. They all work under the coordination of Rorokan Jero for internal affairs who coordinates directly with the chief. The installation of the micro hydro turbine started in 1997 in the Cicemet hamlet where the community worked together to build the micro hydro which was upported by the Institut Bisnis dan Ekonomi Kerakyatan (Yayasan IBEKA).

**Janet:** I have read that the traditional rice ceremony, Seren Taun (Komara et al. 2022) which is the core tradition also provides the basis for educating the young people on agricultural techniques and is in line with the Indonesian ministry’s approach to supporting Indigenous wisdom through ethnopedology. The Ministerial Regulation No. 69/2013) stresses the importance of building on indigenous wisdom through ethnopedagogy (Komara et al. 2021). This resonates with our research findings on the importance of building on Indigenous wisdom^13^ along with gender mainstreaming. One of the areas that could be addressed include the health related problems associated with female circumcision, a practice that is commonly applied in many parts of Indonesia (Kine 2016). Kine states that at the time of reporting between 2010 and 2015 almost 49% of women had been circumcised. Soemarwoto (2011) refers to circumcision as a central practice for males and females in Kasepuhan. Gender mainstreaming is required in terms of Indonesian Law (see Guritno 2019) who cites the People’s Consultative Assembly (the highest political body in Indonesia) Decree Number IV/MPR/1999 on the Broad Guidelines of State Policy 1999-2004. In Ciptagelar Gender Mainstreaming is interpreted in terms of recognising the complementary roles of men and women. This is also expressed in ritual terms as gender equity expressed in ritual dress which indicates women's special relations with humans, God and the environment.

This is indeed a hierarchical community governed by traditional norms and there is scope for improving equity, based on collaboration with Abah Ugi (the chief) and the door is thus open for further engagement on exploring the notion of equity. Women have a voice in public discussions and rituals. Men and women have roles which could be discussed as to whether work should always be defined along gender lines.

### Communication and Links with the Outside World

**Ida:** Apart from the effort to fulfil the necessity for effective communication and information sharing, in the long run, the development of local internet infrastructure in the region is expected to support the official state recognition of Kasepuhan Ciptagelar indigenous rights. This approach is also expected to increase deeper understanding of customary law that is in line with conservation and development of local culture and tradition, as well as the protection of the forest area in the region. In this particular context, internet connectivity is also expected to be able to support participatory mapping activities, as well to facilitate data collection and information gathering on the local knowledge, in particular to social, cultural, and regional aspects of the Kasepuhan Ciptagelar indigenous community. The internet network in the region can also be utilized to support efforts to preserve and protect the tropical forest area, including youth and women’s empowerment programs. In the context of the internet utilization by Kasepuhan Ciptagelar indigenous community member, Abah Ugi as the indigenous community leader had expressed a wish for the development of media literacy programs for the youth, women and adults in some villages and hamlets that already having access to the local internet infrastructure. The objective is to ensure that the local residents are able to develop a safe and adequate internet utilization, besides to strengthen the traditional social structure, local values, political participation, and cultural resilience in the region. Apart from plans to conduct regular media literacy initiatives, it is also expected that the internet may be utilized to support the efforts to deploy environmental monitoring and protection, particularly for the forests area in surrounding the Kasepuhan Ciptagelar region, in which are also being protected by customary law. Environmental monitoring may be conducted through remote sensors that are linked to the local internet infrastructure. In this case, the data and information that is collected from the remote sensing devices may also enrich observation of the traditional agricultural patterns that have been preserved for generations. In the larger context, this approach can also enrich the local knowledge, as well as to the climate change adaptation and mitigation process. Some aspirations on how the internet connectivity should be used by the community member of Kasepuhan Ciptagelar is expected to lead to further ideas and interests on how the internet may give benefit to the local knowledge production and distribution, as well to support the learning process in the broadest sense. In this context, the development of local community-based internet infrastructure in Kasepuhan Ciptagelar region is expected to not only open up learning opportunities on how to make use of information and communication technology (ICT) in rural areas but also supporting knowledge production and distribution on related issues to food sovereignty, climate change mitigation and adaptation, as well as the collective effort in the implementation of sustainable development goals (SDGs).

## Findings and Analysis

The discussion is organised in terms of key concepts suggested by Elinor Ostrom. An appreciation of this wisdom is beginning to be understood in the works for example of Stiglitz et al. in Mismeasuring our lives in which thy make a case for protecting wellbeing stocks, which make up the fabric of life, instead of aiming for profit, we need to aim for re-generation of systems and protecting or sustaining wellbeing stocks (Stiglitz et al. 2010) through protecting many, diverse species and through understanding that communication is a multispecies endeavour (McIntyre-Mills 2021a, b, c, d, e, f) Let us discuss each of Ostrom’s 8 principles which appear in bold italics below:

We need local management to address the following issues explored by Ostrom.^14^

**Janet:** At this point I will sum up that the commons in Kasepuhan Ciptagelar refers to the land which is protected by a shared culture that has remained intact for over 600 years. Although some of the rice fields are managed by specific families, all the harvested rice is stored and available to the community. If people are in need then they can draw on the reserves from the rice barns. Rice is not commodified or sold and all the planting, managing of the crop and harvesting is conducted communally according to a set ecological calendar. In other words all the farmers do the same activities at the same time.

Families fill their family rice barns which are available to those in need. The more rice they have, the greater the status of the family, because they are in a position to help others. This is quite different from wealth based on profit.

**Ida:** Ciptagelar indigenous community in Southern Banten represents a semi-nomadic indigenous community with a communal customary land tenure type. Researchers found that the community has moved 11 times in more than 1,300 years. They live in 68,000 ha of the Halimun Salak National Park since 1902 (Abdulharis et al. 2007). Until now, the Ciptagelar indigenous community did not have legal ownership of land (Kusdiwanggo 2014a, b; Wisudawanto and Abdulharis 2008) who explain that the forest (as a common resource) is classified by three types spanning: a ‘Forbidden forest’ for conservation, an entrusted forest for non business community needs such as herbs and dead branches and a so-called ‘Production forest’ for more regular daily usage. Other common goods in the conservation area also include the land, rivers springs and rice fields . The agricultural system generally ensures the practice of sustainable farming guided by the *local indigenous knowledge system*. Farming follows a cultural practice: everyone will start working together in the chief’s land, then they work on their own farm. None of the community members are allowed to receive payment when they work on a farm. The distribution of harvested rice is collected from each household and stored in *the Leuit Jimat*, which means ‘the magical rice barn’.

### Ostrom’s Eight Principles

Janet: Let us discuss the principles in terms of the social, cultural and environmental context of this region?

- ***Principle 1 1. Commons need to have clearly defined boundaries. In particular, who is entitled to access to what? Unless there’s a specified community of benefit, it becomes a free for all, and that’s not how commons work.”***

**Ida:** They respect nature and the universe in the same way as they respect their parents which shapes all aspects of the way they organise their community.^15^The notion of ‘Mother Earth, Father Universe’ *“Ibu Bumi, Bapak Langit’* is reflected in a saying *“Jagat Leutik, Jagat Gede - Jagat leutik sanubari, Jagat gede bumi langit”* (Mother Earth, Father Sky ’is reflected in a saying“ Small World, Great World - Small World of Heart/conscience protects the Big World of Heavenly Earth ”

**Janet:** When the chief ‘listens to nature’ or makes contextual decisions to protect the forest, does he draw on ritual and traditions which act as a form of memory code (Kelly 2016) to carry knowledge in their oral history? Iskandar (2021) also talks of the importance of water governance for irrigation and about the use of new electrical turbines which are introduced in such a way that the core values of the traditional rice culture is protected.

**Ida :** The community of *Kasepuhan Ciptagelar* is spread across three districts around the border area of Banten Province and West Java Province. It includes the Lebak Regency, Bogor Regency and Sukabumi Regency. The term ‘*Kasepuhan’* means the elders, referring to a traditional system of leadership. *Kasepuhan Ciptagelar community* members called themselves the descendants of *Pancer Pangawinan .*

The community has important three pillars of human relationships, the 'Pancer pangawinan' as human beings should embrace humanity, 'ngaji diri' (self-awareness). This becomes an important value to care for other beings, and the member of community should consistently practice “*ucap jeung lampah* “ the intention of speech should comply with daily behaviors. Honesty has a high cultural value, the term ‘*mipit kudu amit ngala kudu menta’* for example teach people that we need to ask for permission for every action, we need to pray for blessing, safety, and success when picking and harvesting.

The community extends across the Halimun Salak National Reserve, Cisolok Sub-District, Sukabumi Regency of West Java. Overall the area near the border of Bantem Province consists of 586 hamlets comprising the Ciptagelar village.^16^

The Kasepuhan Ciptagelar village has distinctive characteristics in terms of its remote location and settlement in West Java which has been widely documented.^17^ The village has its own radio and TV (Tarina et al. 2021) hosted by talented local residents and led by Abah Ugi who holds an electronics degree.

**Fig. 1:**
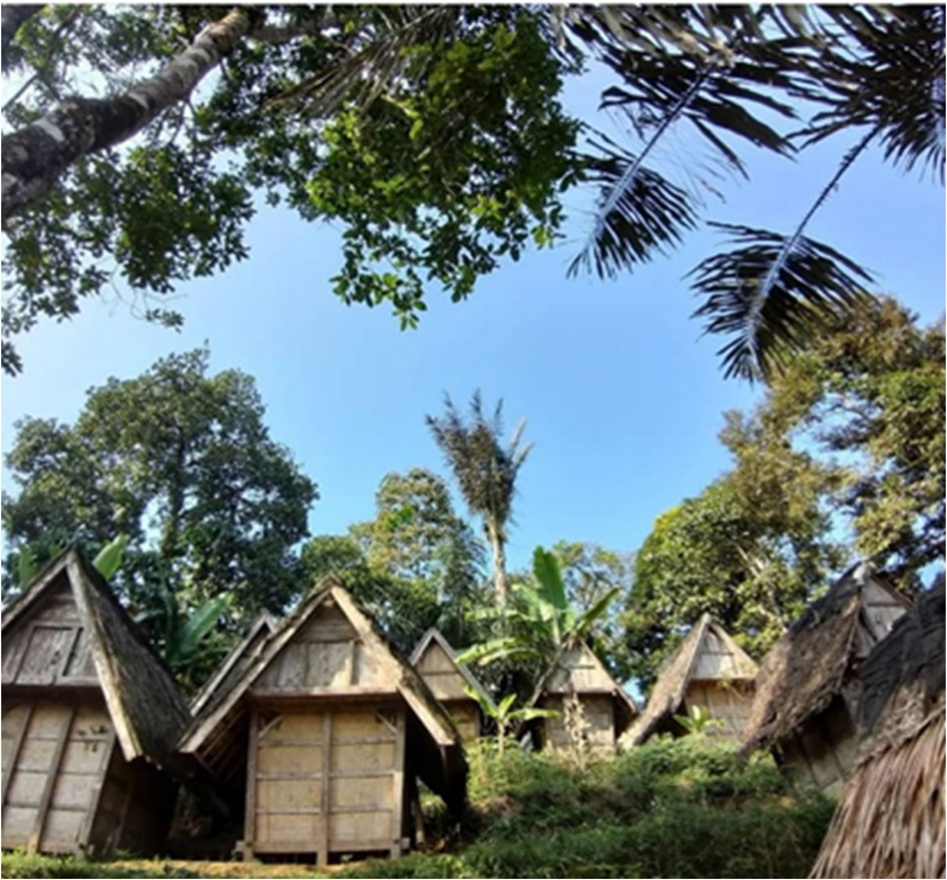
Each house has its own rice storage barn, known as a ‘Leuit’. Source: Ida Widianingsih 2019

**Fig. 2:**
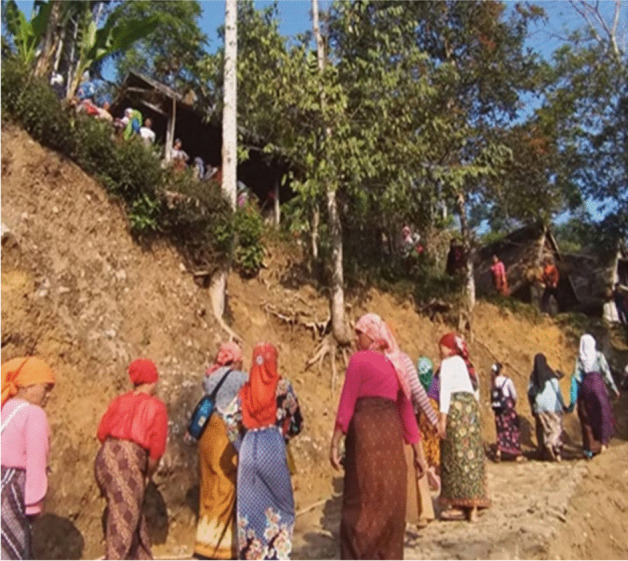
1700 women of different ages work together to grind 1400 bunch of paddy into rice beads using traditional tools made of old solid timber. They worked in 15 different *lisung* (traditional buildings for rice processing, such as the one above. (McIntyre-Mills et al. 2019)

**Fig. 3:**
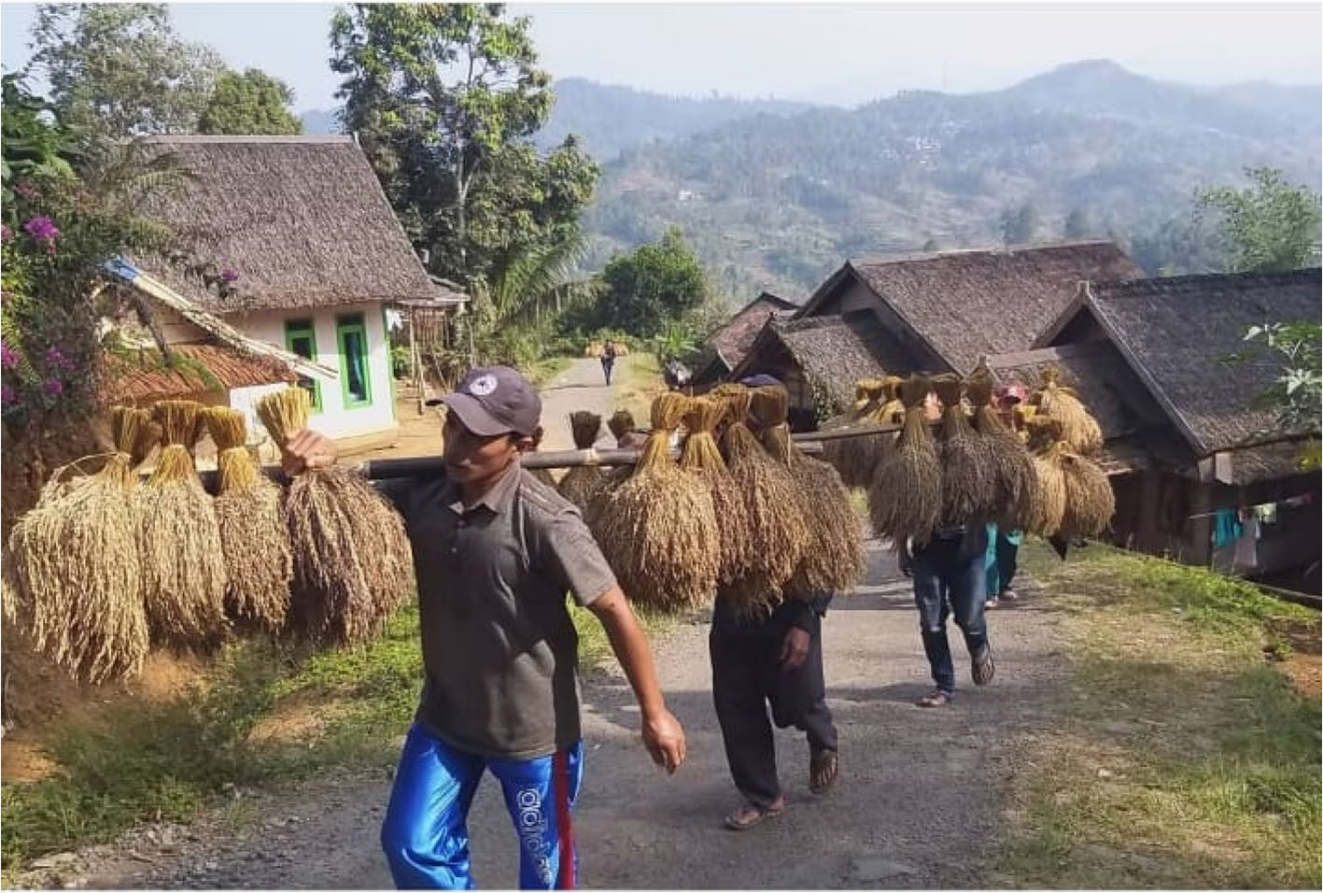
Men carrying the paddy from their own village as a gift to the chief for the Ponggokan ritual. (McIntyre-Mills et al. 2019)

Houses in the village are made of wood, covered with bamboo, and roofed with dried palm fronds. They all live sustainably. Each house has electricity and is built according to a specific plan to ensure that the house does not rely on foundations in the ground. The architecture is very specific. The structure is wooden and rests on stones. No living beings should be under the ground. It is believed that only the dead should be sheltered by the earth and all the materials used should re-generate. They have an open space in front of the house and they leave their shoes outside (Figs. 1, 2 and 3).

**Fig. 4:**
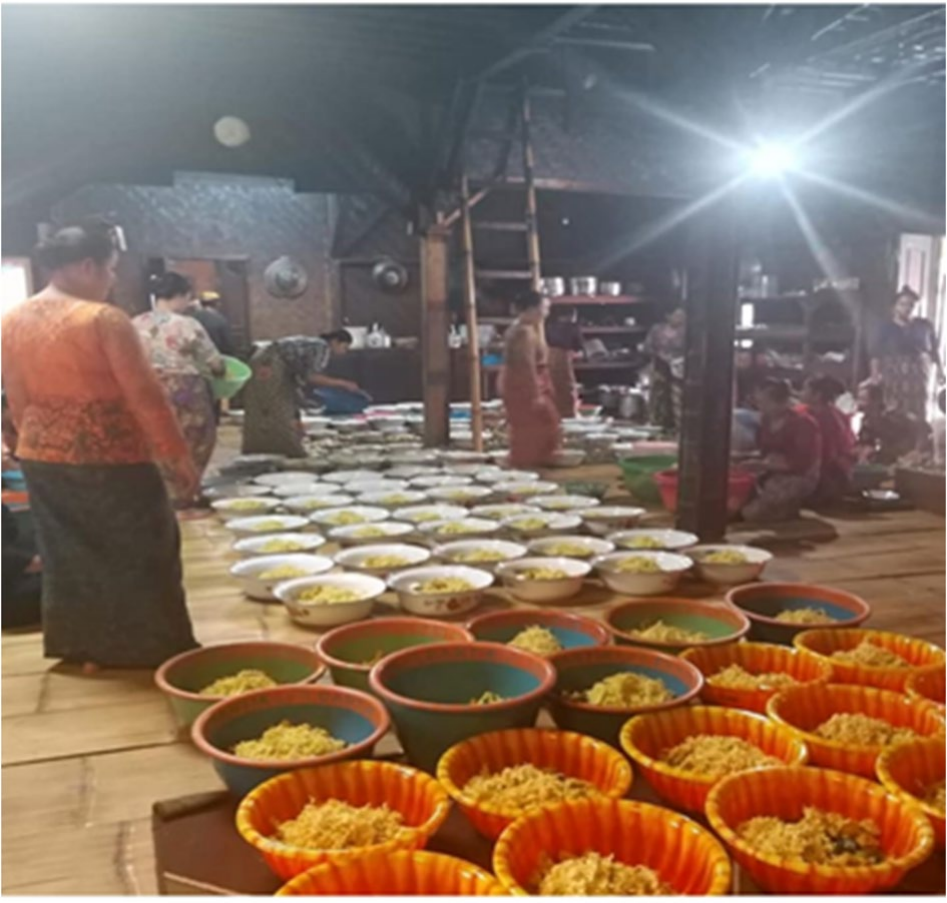
The rice preparation for a communal feast (McIntyre-Mills et al. 2019)

**Fig. 5:**
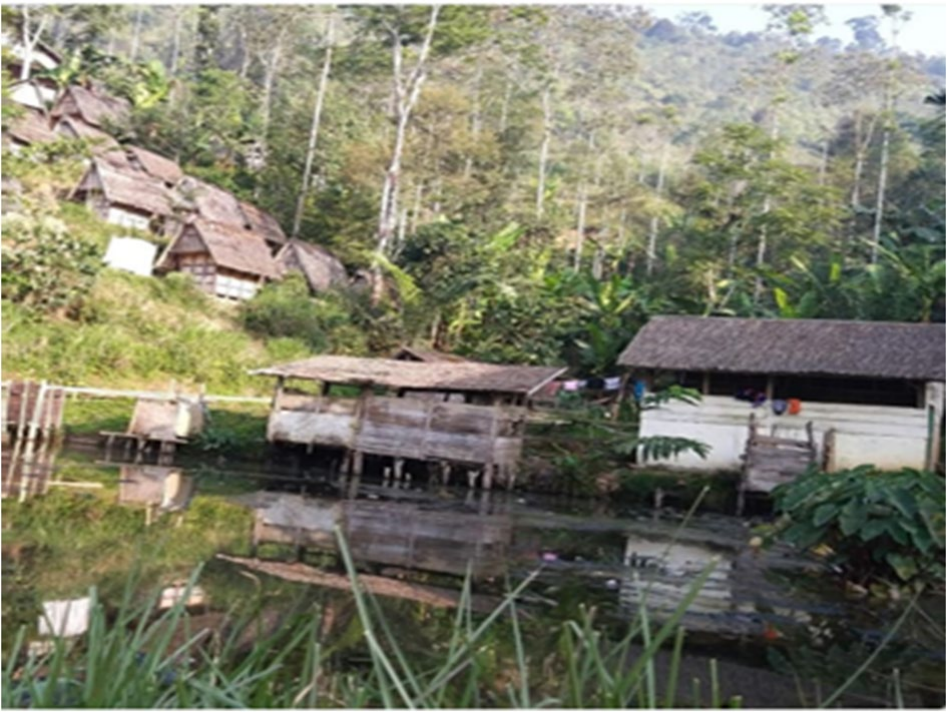
Typical fishpond in Ciptagelar village with a traditional latrine and bathroom. In the background are the rice barns (McIntyre-Mills et al. 2019)

**Fig. 6:**
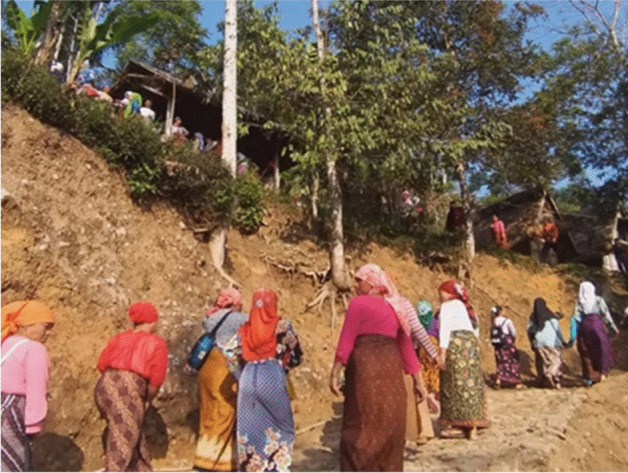
1700 women with different ages work together to grind 1400 bunches of paddy into rice beads using traditional tools made of old solid timber. All of the women also actively participate in grinding the paddy (McIntyre-Mills et al. 2019)

**Fig. 7:**
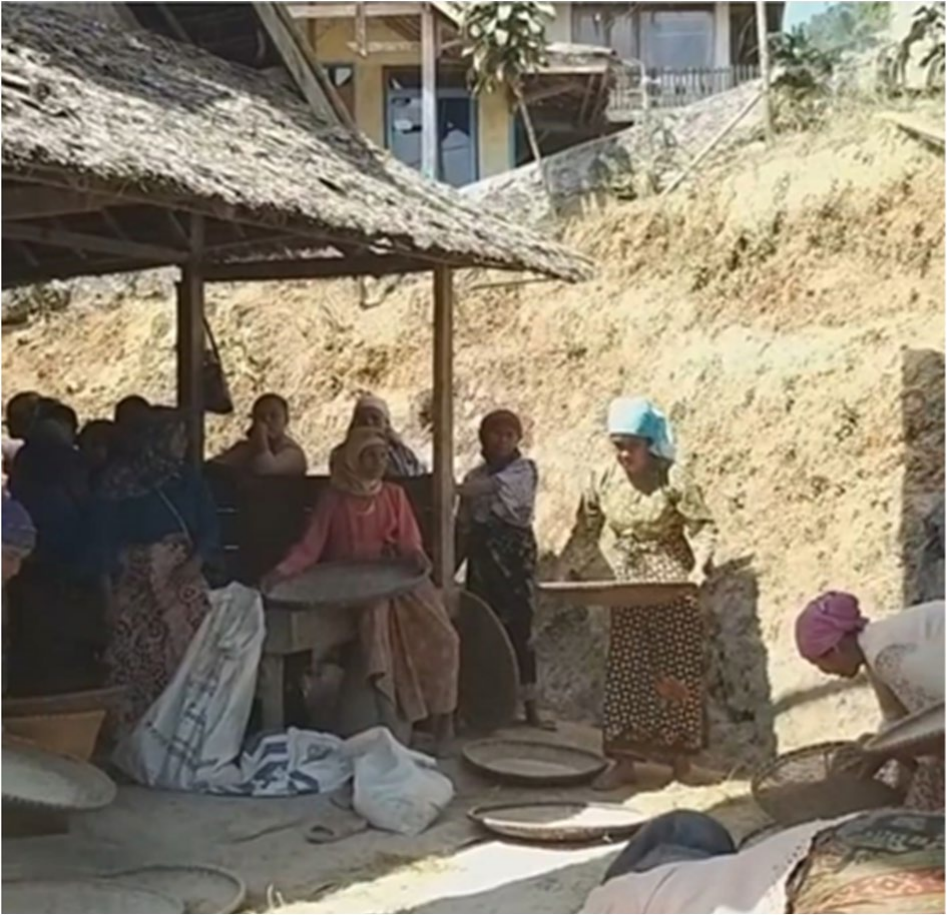
The preparation of nasi kebuli, some women cooked in the kitchen of Imah Gede (The big house/the palace) under the coordination of Jaro (photo supplied by Widianingsih, 2019)

**Fig. 8:**
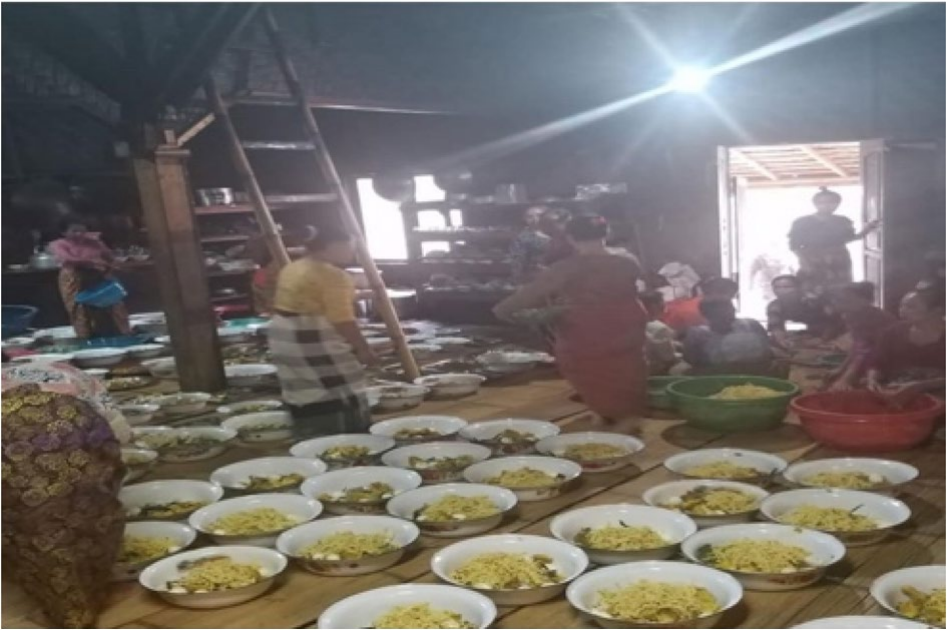
The wife of the Chief manages the whole cooking process (McIntyre-Mills et al. 2019)

After harvesting, the paddy is transported to the village and Leuit. It is a core activity around which their society revolves. The Kasepuhan Indigenous community recognises three type of leuit is the magical rice barn (Leuit Jimat), the traditional/elder rice barn (leuit rurukan (adat), and community rice barn (leuit warga) (Kusdiwanggo 2015). The number of leuit rurukan and leuit warga keep increasing every year because it is an integral part of the settlement and cultural spaces (Figs. 4, 5, 6, 7 and 8).

The Kasepuhan Ciptagelar Indigenous Community only plant rice for daily consumption, it is forbidden for them to sell or buy rice because rice is the Goddess Nyi Pohaci Sanghyang Asri or Devi Sri.

Paddy or rice is sacred to the community. Women have special place in Ciptagelar community, most of rituals give a special space for women (Kusdiwanggo 2014a, b, 2015, 2016a, b) to reflect their social and governance roles. Gender equity is also expressed in ritual dress which indicates their special relationship with humans, God, and the environment (Astutik et al. 2018).

**Fig. 9:**
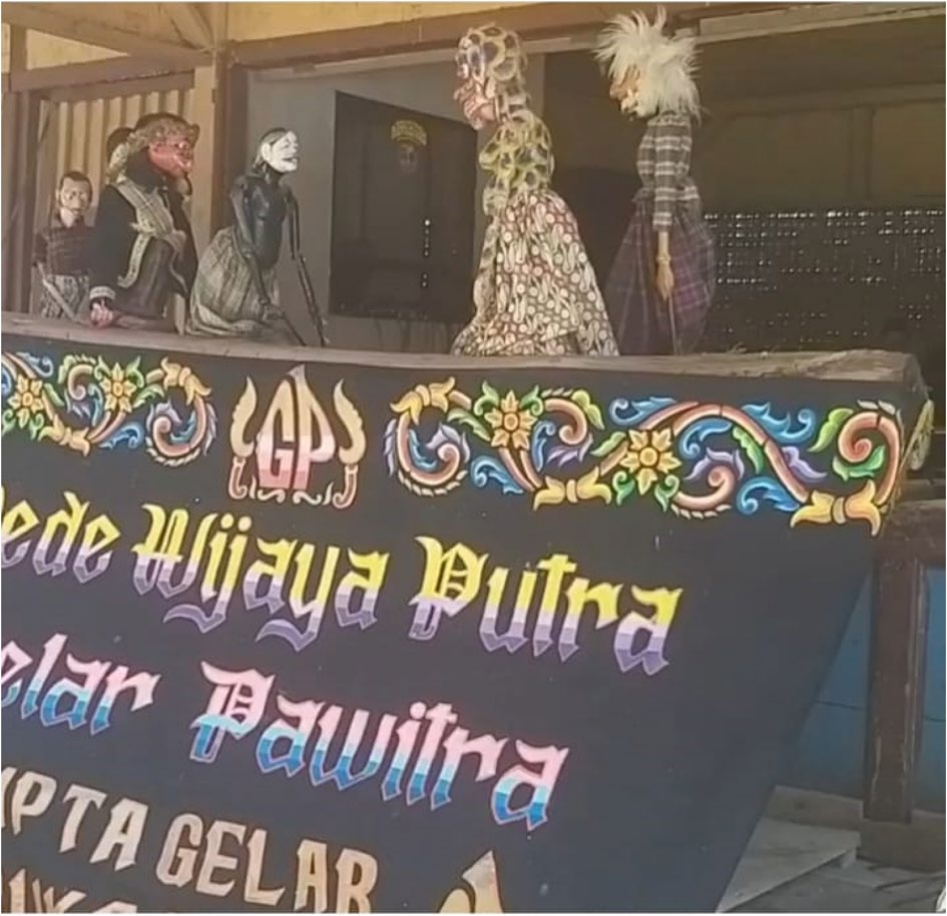
Traditional puppet show depicts historical and mythical events (McIntyre-Mills et al. 2019)

Interviews with the chief and village elders revealed that they have moved about 19 times under 11 different chiefs. They hold a strong tradition, abah (father) is other name for the chief, the community leader. Abah would works with his wife (emak). The chief also supported by village elders *Rendang Kande* and village government officials called *Rorokan*. The *Rorokan* have distinctive responsibilities. For example, the members of *Rorokan Padukunan* are responsible for leading all traditional rituals, whilst the members of the *Rorokan Pamakayaan* are responsible for all agricultural activities (Iskandar 2018, 2021, see also Yeong Ran Suh 2021 on rice and ritual) (Fig. 9).

The Kasepuhan Ciptagelar community has practiced Sundanese culture and philosophy for centuries and researchers stress that rice is central to their cultural beliefs. The community has three important pillars of human relationships, namely the *'Pancer pangawinan' *which means that as human beings we should embrace humanity, '*ngaji diri*' (self-awareness) becomes an important value to care for other beings, and the members of community should consistently apply their values through their speech and actions. Honesty has a high cultural value and the term ‘*mipit kudu amit ngala kudu menta’* , for example teaches people that we need to ask for permission for every action, we need to pray for blessing, safety, and success when picking and harvesting.

The term “*mupusti pare lain migusti*” embraces a deep meaning about the way people value rice and perceive it as sacred. Their culture forbids the commodification and sale of rice, because rice is the Goddess called *Nyi Pohaci Sanghyang Asri*.

The Ciptagelar community has a strong cultural connection with rice reflected in at least 32 cultural rituals within one rice planting cycle. The traditional farming system is carried out once a year with the concept of Mother Earth, Father of Heaven and Master of Prey.

The Kasepuhan community plant a local grain called *'pare ageung'* (big Paddy) which consists of at least 100 species, according to some research, only 50 species of the *'pare ageung'* are used. The classification of the paddy is based on external morphology such as the colour and the shape of the grains, and the presence of fur at the tip of the grain. Within one cycle of rice farming, there are 32 cultural rituals called, *ngabaladah* comprising many practical steps (drawing on Putra et al. 2019) which includes:

Weeding the fields , rites of gratitude, soaking and filling the land with water, marking the location that will be used as farm, cleaning the land, usually for 1 week after drying the grass from two weeks to a month, making rice seedbeds by spreading rice , burning dry bushes for fertilizer, gathering the remains of unburned bushes), burning the remains of bushes that have been collected),softening the soil), planting rice seeds, weeding to protect the crop, planting rice),Weeding out the grass), Cleaning the weeds in the fields.

Rice farming is a way of life. Every family in the *Kasepuhan Ciptagelar* community has their own fields and barns (*leuit)*. The male members of the Kasepuhan Ciptagelar required to wear "headband (*iket*)", it is a symbol of unity reflecting in the term ‘*saiketan sabeungkeutan’* (Putra et al. 2019).

The philosophy of life of Ciptagelar indigenous community is both communal and dedicated to protecting nature by working in harmony.

As mentioned previously, the chief of Kasepuhan Ciptagelar is appointed based on family geneology known as ‘sesepuh girang’ (traditional chief) called Abah Anom. The chief is supported by respective elders (baris kolot). Abah Ugi recently remarried,^18^ as in terms of their customs the leaders need to be both male and female to achieve balance and harmony. Interestingly, the traditional leadership structure is continuing to evolve responding to the dynamic of social life and the adaptation to new knowledge and technology.

**Janet:** It also seems as if the village moves according to a cycle overseen by the chief so that the forest is protected and so the villagers are semi nomadic. How long do they stay in a specific location? Please explain the rotational cycle – to ensure that the forest and surrounding agricultural lands are rested?

**Ida:** Yes, the community is nomadic and they move from place to place following a specific plan or pattern that is directed by the Chief according to his spiritual inspiration or wangsit which he inherited from his father. The first item to be moved is the rice storage known as “leuit jimat’ (magical rice Barn) in which rice can be stored for up to 60 years. Wealth is measure by the amount of rice you have, but it is not commodified or sold. *Kasepuhan Ciptagelar* is formed based on the moving of the Chief (*Sesepuh girang)* from *Ciptarasa* village in Juli 2000.

The name of Ciptagelar was taken from the chief’s name ‘Encup Sucipta’ or ‘Cipta’. The moving process is called *hijrah *wangsit* –* the calling from the ancestors (*karuhun),* the Chief will receive a vision *(Uga)* that will provide the basis for his decisions. The nomadic cycle is thus not perceived to directed by dreams, semedi or special rituals which the chief is able to interpret. Every time they move, they carry the magical rice barn (*leuit* si Jimat), *pangkemitan, pangnyayuran* and *ajeng* wayang golek. This process is guided by oral history passed from one generation to another.

The Kasepuhan Ciptagelar Indigenous community is part of the Pajajaran kingdom in Bogor, in the 16^th^ Century, but the kingdom was taken over by Banten Islamic Sultan under the leadership of Sultan Maulana Yusuf. It was the last Hindu kingdom in Sunda land (West Java). The king of Pajajaran kingdom- Prabu Suryakancana or Prabu Pucuk Umun was the last king of the *Pakuan Pajajaran* who told his ministers to save and protect ‘magical tools’ known as *barang-barang pusaka*.

The king and his followers were evicted and fled to Palasari—Pandeglang, Banten, while his ministers went to Jasinga Bogor, then moved to Lebak Binong in the Lebak district of the Banten province. They kept moving around the Salak mountain National reserve to hide from the Banten Islamic Sultan. In 1957 the centre of the *Kasepuhan*, moved to Cikaret (Sirnaresmi), then to Ciganas (Sirna Rasa) in 1972, Lebak Gadog (Linggar Jati) in 1982. In 1983 they moved to Datar Putat (Cipta Rasa), and settled in Cikarancang (Ciptagelar) in 2000 where they are currently based.

**Janet:** Thank you for sharing this history. So to sum up, the community was founded by people fleeing into the forest for refuge. Please tell us more about this system of governance. You mentioned that the Chief has final authority in both public and private areas of life, as for example before a marriage, the king needs to be consulted and before any changes are made in the community the king needs to ask permission from the forest, land and waters?

**Ida:** The king and the community try to follow nature’s laws as they consult a traditional calendar that has been handed down as a form of oral tradition. Because the community strives to protect their core values (Ramadhan and Suryani 2018) all developments remain in line with their approach to protect the environment as a community. I also note that no changes are made to the rice growing cycle, other than ensuring the water supply.

**Janet:** Thank you, for this explanation. Let’s consider Ostrom’s second principal:

- **Principle 2 “Rules should fit local circumstances. There is no one-size-fits-all approach to common resource management. Rules should be dictated by local people and local ecological needs.”**^19^

**Ida:** The relationship between people and nature is not debased through trading. Rice is regarded as a goddess and the forest as a living being with whom the chief communicates. Ciptagelar indigenous community believe in the harmony of human and nature, nature communicates with human through some readable signs. For example, the practice of agriculture and forest managements follow the principle of is ‘*ngereut jeung neundeun keur jaga ning isuk’*, meaning that we have to spare/save for the future. The traditional barn or *‘leuit’* is a compulsory building owned by a family. The modest lifestyle shown in the principle of *“Saeutik mahi, loba nyesa, halal didaharna’* means that little harvest should be enough to share, for saving, and properly eaten (Putra et al. 2019)*.*

In terms of forest management and environmental preservation, local wisdom and community customs are the core values. For this, the forest is divided into 3 types or zones, namely *leuweung titipan*, *leuweung tutupan*, and *leuweung Garapan:*

Janet: Astika (2016) sums this up as follows:

“The management of the forest environment cannot be separated from their local wisdom and customs. Based on the local customs, the forest has been categorized into three types, namely forbidden forest, cover forest and cultivation forest. The zoning of the forest represents the form of their local wisdom in their efforts to preserve the forest environment.”

Iskandar (2018, 2021) also stresses that the success in achieving balance is a result of ensuring that there are sacred, areas that can be harvested and areas for cultivation. The sacred areas are zones that are vital for protecting ground water and contain the sources of springs

**Ida:** Even though the Kasepuhan Ciptagelar community live in a remote region, they are able to keep up with the development of information. This must be related to the proverb "*mun teu nyaho kudu nyaho*" –If you don’t know, you have to know. There is also a saying that ‘they must be able to keep up with the times, but do not get carried away".

Community members are obliged to maintain cultural values and adapt into new technology development. Under the leadership of Abah Anom, the introduction of electricity was started in 1985–1988 through the installation of a generator with 3,000 watts of power for Cipta Rasa village (Desi and Tarina (2021). The recent chief, Abah Ugi is open to new technology, he uses drones to oversee the Kasepuhan Ciptagelar area (Kompas TV documentation, 10 June 2015).

**Janet:** Thus traditional customs and values are upheld and technology serves these values.

Let us discuss Ostrom’s third principle^20^:

- **Principle 3 “Participatory decision-making is vital. There are all kinds of ways to make it happen, but people will be more likely to follow the rules if they had a hand in writing them. Involve as many people as possible in decision-making.”**

**Janet:** The governance process involves community participation, but to what extent can people shape decisions?

**Ida:** The rice ceremony, Seren Taun (Komara et al. 2021) is central and oral history remains important along with the arts (dance, music song) along with visual arts and crafts. This community is able to document its history using multimedia through its own radio and TV station which was set up as the leader of the village has ICT skills and is a qualified engineer.

**Janet:** This seems to provide opportunities for so-called ethnopedagogy to flourish as elders share their knowledge and previously you explained that some of the younger residents help with the new technology. The customs and rules associated with the rice ceremony are carried in song, ritual and dance and they are sacred and unchangeable, but nevertheless there seems to be adaptability around introducing technology to serve the commons. I am also interested to see how the traditional puppet shows complement modern forms of communication technology to support education.

Ida, please tell us more about the TV network and the extent to which oral history is being supported by literacy and numeracy? Please also explain how the meetings are  organised. What is their frequency and timing with the entire community?

**Fig. 10:**
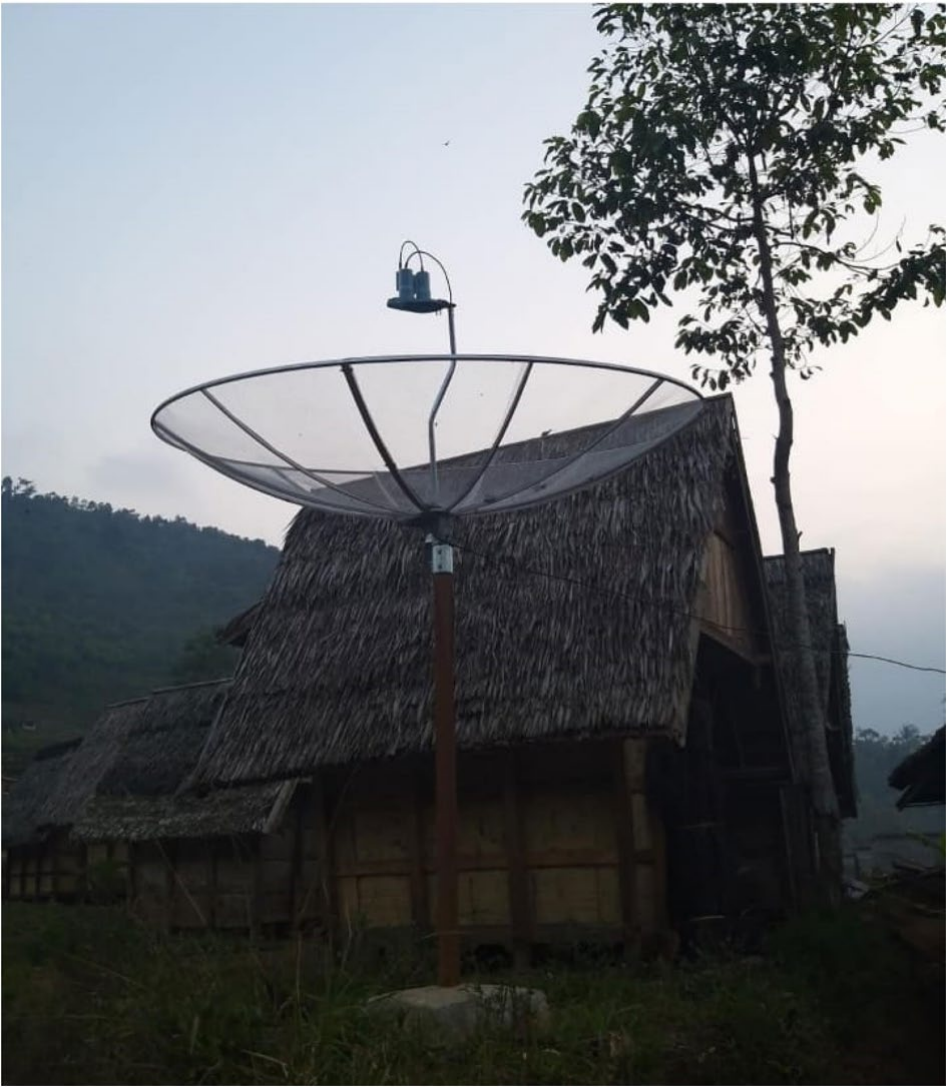
A Television antenna has been raised near the traditional rice barn behind a house (McIntyre-Mills et al. 2019)

**Ida :** They have a primary school and a TV station for public education (Fig. 10).

Universitas Padjadjaran collaborates with the Common Room Networks and we have been involved in helping to make some of the programs to train young people and women group in sustainable agriculture through a comparative study. The CigaTV is used to help train and educate the villagers. Governance is centred around management of agriculture and the kitchens where cooking of rice meals is supervised to provide for 3000 to 4 000 people. Each step of the process is carefully supervised so the chopping preparation is done together and then the cooking is done at the household level by each participant. The level of organisation is complex but governed by traditions that everyone follows.

Without any form of digital communication the leader in charge of agriculture and food was able to organise a community gathering of about 3000 people and to organise a meal for this large group that met for governance purposes, to hold a kind of census.

If young people show interest and aptitude they are encouraged to continue their schooling outside the community.

The young chief/king has an interest in digital engagement and has also appointed a leader to address digital engagement. When we helped fund a tower to enable internet access the chief led a ceremony to ask the gods of the forest, land and water if putting up a tower was a good idea.

Traditionally the members of the community ask permission from the king and the land before making any changes and they also consult their traditional calendar. For example,when they plant rice they hold a ceremony and worship the goddess of the rice. They carefully preserve local seed diversity and have many different coloured grains. When the rest of the region’s farmers suffered from drought, the farmers in this area did not, because their grains were more resilient.

They focus on ways to work with nature such as how to prepare organic fertilizers by storing manure for six months that is mixed with other organic materials (leaves, trash, trunk bananas, rice, furnace ashes, etc.). This is then used as fertilizer in rice or vegetables in the garden.

They also learn how to harvest and store rice in an effort to overcome food shortages. The storage units are placed in groups to enable better management and security. The students learn that preservation of rice seeds begins by doing good seed selection of rice based on an understanding of seeds. This knowledge is based down from one generation to another so that young people learn the criteria for selecting seeds. In this area the local rice varieties are white (locan, sari kuning and jaulang) and red (gantan and cerai) and the pattern of planting is to plant rice only once a year with a view to maintaining the level of soil fertility.

The use of rice straw as an organic fertilizer is based on cutting rice straw after the harvest, then the straw is stored in an area so that it can decompose.

The students learn that each head of household is obliged to save rice in the communal barn (leuit si jimat). This practice ensures that those in need can borrow rice from the barn.

All the stages of agriculture are communal and based on customs honed through generations of trial and error. As a community they engage in simultaneous planting and they work together

**Janet:** I see from the chapter by Soemarwoto 2011) that unlike other farmers who rely on so-called high yielding genetically modified rice, they decided to retain their traditional seeds as they are (already) high yielding and also easier to harvest and store. The author explains that they live in harmony with the many creatures in the area and they do not rely on pesticides as they respect all creatures. The following quotation sums up their concept that they should use their head, emotions and body to protect all living beings:

“Kasepuhan recognize that other creatures are dependent on rice in addition to humans. Although Kasepuhan are aware of the damage done to rice by other organisms, such as rats, and of the reduction in yields that results from this, they do not consider them as hama (pests) in the same way as might an agricultural scientist. Kasepuhan refuse to kill them, as they say that these organisms are not causing any harm, only participating in life (ngilu), and anyway will not eat everything. … Kasepuhan tolerate… the rats [that] live off the grain in the rice barns and, in many places around the village, and which are permitted to live freely inside houses, even when they eat food.” (Soemarwoto 2011: 87)

Soemarwoto (2011) concedes that they try to manage the balance through prevention and through only planting once per year so that the agricultural land recovers. Risk is also managed by ensuring that a variety of grains are planted and that crops are planted and harvested at the same time. Through empirical experience they know when particular insects are likely to be most prevalent and they take appropriate responses. This community has managed to raise productivity whilst avoiding pesticides and by using local seeds that they have nurtured over many generations, so the indigenous wisdom is protected, respected and passed on To guide the types of rice (landraces) to be planted. Soemarwoto (2011: 91) explains that decisions are guided by: Through the notion that:

“… the pancer-papadon relation of five symbolic parts of the human body i.e. the pancer (trunk) and the papadon, the head and the limbs. Thus rice plants must be selected carefully in order to obtain a balance of affective, cognitive and practical qualities. …”.

To a large extent the community (although highly participatory) adheres to traditional rules for living and in many ways the decision making is hierarchical, albeit expressed in terms of natural law and the leaders mystical understanding of what is best for the community.

**Ida:** Other farmers in West Java region use GM seeds introduced by the government during the agrarian revolution under Soeharto government, but according to Iskandar (2021) this area has had higher yields based on local traditional agriculture.

**Janet**: let us consider Ostrom’s fourth principle^21^:

- **Principle 4 “Commons must be monitored. Once rules have been set, communities need a way of checking that people are keeping them. Commons don’t run on good will, but on accountability.”**

**Janet:** Please explain how the calendar is used to project and monitor the commons.

**Ida:** The sacred forest protects the water table and the system of governance has been handed down through Sundanese oral culture for 350 years. Their approach to protecting food security is linked with religion and ritual linked with a calendars. The calendar is accurate and is governed by the sun and the stars and linked with daily life and agriculture.

The late Suhardja Adimihardja (February 2016) from Bandung Institute of Technology studied the calendar and said it was very accurate. The indigenous knowledge in creating a natural balance of the environment comes from the idea that humans could take benefit from nature, but also have to preserve it.

The Indigenous People of Kasepuhan Ciptagelar only cultivates their land once a year to respect the land. The Mother Earth as a living creature. It is an obligation for them to show respect through traditional ceremonies during the cultivation period. Furthermore, the *Guru Mangsa* (the universe) and the constellation of stars *(kerti* and *kidang)* at night becomes the compass to determine the cultivation schedule. The goal of farming is *ngudag akuan* (pursuing the land rights). The *kerti* star is an early sign to work on the land, even though the rain has not yet fallen. The indigenous community use a proverb “The date of *Kerti turun beusi*, and the date of the *Kidang turun Kujang* (a traditional Sundanese sword)” when the *Kerti* star appears on the western horizon, all the *kasepuhan* Ciptagelar community have to prepare their farming tools (made of iron).

When the *kidang* star appears on the eastern horizon, it means that everyone should be ready to clean the land for farming (Adimihardja 1992 who explains the seasons and the related agricultural activities^22^). According to the local community, the months of September to April are for faming while the months of May to August is the time for other creatures who are not regarded as so-called pests at other times of the year, so that they have time to flourish.

**Janet:** Please discuss the role of Master of the Pests. Perhaps the role is to protect biodiversity? Perhaps this would be a better translation? The notion that other species have rights seems to be fulfilled by this cycle?

**Ida:** Yes, they don’t use pesticide nor modern technology. There are five constellations of the stars as a reference in managing arable land and agricultural activities:

They also practice food security monitoring through local traditions called *ngadiukeun indung* (Settling the mother) to protect the growing cycle (Khomsan, Riyadi, arliati 2013 also see Wahyu, Kusdewanggi 2012). It is forbidden for *Kasepuhan Ciptagelar* community to sell and buy rice, selling rice means selling their dignity. They believe that rice is the Goddess *Dewi Sri or Nyi Pohaci*, the symbol of welfare and fertility. The blessing for Pajajaran kingdom community. The rice also comes from earth, the same as human that created from earth.

Abah Ugi (the recent Chief and the 11^th^ generation of *Kasepuhan Ciptagelar* community) has explained that the paddy in Ciptagelar villages can only be used for local grains that have been passed from their ancestors. The way *Kasepuhan Ciptagelar* community planting their rice is different with those farmers in in other villages in West Java.

Even though the *Kasepuhan Ciptagelar* community are quite open to the introduction of new technology, they do not make any changes to rice cultivation and utilization. The Rice cultivation system is also a model of regenerative education, those who are married are obliged to cultivate rice by following all the existing rules. As stated by *Rorokan Pamakayan* (Minister of Agriculture) that “*Mun teu digarap, bakal muuk, ngabadi, ngageringan*” (If a married citizen does not cultivate rice, it will hurt the person in the form of illness) (Budiaman et al. 2021))

**Janet:** The notion that rice is sacred is similar to the idea that pumkins and finger millet are sacred in Venda, South Africa. The community in Ciptagelar take this further by stressing that rice cannot be commodified and sold. It is regarded as a life force. This is a very important difference between Western capitalism as agriculture is managed through a careful process carried in ritual

**Ida:** Yes the protection of rice requires managing the agricultural environment, the settlement and the way the villagers interact with the environment citizens. The protection period is held during the second stage of the planting period. The protection is usually called ‘sawen’.

The rituals are both practical and spiritual. According to myth, the paddy was born together with its pests, both microorganisms (Kalabuat) and animals (Budugbasu), so no pure paddy was free from disease. Sakuren, means they always coexisted from the very beginning and will continue in harmony forever. The Pager ritual protects by diverting without destruction; thus, paddy and pests are harmonized.

Janet: Thanks for sharing your detailed notes on the rituals using bamboo rods and explaining that for example, “Cangreud Sulaiman is invoked, because he was the apostle who could communicate with subtle creatures.” The details of the rituals need in depth discussion as they carry a great deal of wisdom, both practical and spiritual for the harmonious co-existence of the rice crop and other creatures.

- **Principle 5 ‘ Sanctions for those who abuse the commons should be graduated…”**

**Ida:** In this community, the wrong doers are subject to customary law which is based on the notion that if you do the wrong thing, then you will receive justice from nature. It seems that the customs are imbued in all aspects of life. The community however also follows Indonesian law and those who break the law are handed to the relevant authorities. Abah Ugi, explained that there would not be any punishment of criminal acts against the indigenous community. The verdict will be handed over to the authorities for trial. In the case of conflicts break amongst the *Kasepuhan Ciptagelar* community, it will be a traditional conflict resolution process. The oldest person in the family (*Rendangan*) will discuss the solutions of the problems. If no agreement concluded, the chief will call for a meeting of the respective *rendangan* and every single head of the village. *Kasepuhan Ciptagelar* community has no written customary law, instead they are practicing an oral customary law through a regenerative process. The notion of “*pamali*" often use as a “NO sign”. Those who break the customary law will be punished by spiritual power (*kabendon),* whoever violating the ancestral guidance will be misfortune (Adimihardja 1992). The concept of *kabendon* also apply to those commit a murder, the nature will punish the person and will get punishment. The verdict will feel like the person has ‘died even though the person is alive” is what this proverb means "*mitilas kadepna, legok ku tapakna, calaka ku amal amal-amalana*".

- **Principle 6 “Conflict resolution should be easily accessible. …..”**

**Janet:** Local customary laws stress that wrong doers will receive punishment from nature and conflict resolution is based on natural justice and the shame that the perpetrator feels. It has also been confirmed that criminal cases are handed over to the Indonesian authorities. Thus the community has a nested system of laws as suggested by Ostrom and it appears that Ostrom’s final three principles, also apply in this community.

- **Principle 7 ‘Commons need the right to organise. Your commons rules won’t count for anything if a higher local authority doesn’t recognise them as legitimate.’**^23^

**Janet:** In Indonesia local wisdom is appreciated in terms of the law, for instance the Ministerial Regulation No. 69/2013) stresses the importance of building on indigenous wisdom through ethnopedagogy (Komara et al. 2021), but by sharing this case study we can raise further awareness of the need to protect the rights of indigenous peoples and that the way of life and their approach to agriculture in Ciptagelar addresses the concerns raised by the United Nations Agendas, namely:

- *The rights of Indigenous people to protect wisdom and land rights* (see United Nations Declaration on the Rights of Indigenous Peoples (UNDRIP). (2008). Accessed 14 Feb 2016 at: https://www.un.org/development/desa/indigenouspeoples/declaration-on-the-rights-of-indigenous-peoples.html). Nevertheless, Tarina et al. (2021:1009-1010) stress that:

“Article 18B paragraph (2) of the Constitution of the Republic of Indonesia 1945 (UUD NRI 1945) states that the State recognizes and respects the unity of indigenous peoples and their traditional rights throughout life and in accordance with the development of society and principles of the Unitary State of the Republic of Indonesia (NKRI), coupled with Article 28 I paragraph (3) that the cultural identity and rights of traditional peoples are respected in accordance with the times and civilizations.”

- *Reducing risks* by demonstrating ways to live sustainably on the land, thus reducing urbanization and the associated risks (see United Nations, 2014 report on urbanisation; United Nations Office for Disaster Risk Reduction (2015-2030). Sendai Framework http://www.preventionweb.net/drr-framework/sendai-framework/)
- *Enhancing sustainable living* and addressing food security (see United Nations Sustainable Development Goals (2017). https://www.un.org/development/desa/publications/sdg-report-2017.html and United Nations and the 2030 Agenda and the Sustainable Development Goals.
- Mitigating and adapting to climate change (see United Nations (2021). Glasgow Climate Change Conference https://unfccc.int/process-and-meetings/conferences/glasgow-climate-change-conference and
- Finally, the latest IPCC reports^24^ can be served well by learning from Indigenous communities. For instance, the latest IPCC report based on the findings of the Glasgow summit^25^ stresses:

“In the eight years since … the IPCC’s Fifth Assessment Report in 2013, global emissions have continued to rise, temperatures have skyrocketed, and the world has witnessed a terrifying run of extreme weather disasters, from Australia’s 2019-20 summer to the extraordinary heatwaves, fires and floods that have shaken the northern hemisphere this year. In that time our understanding of climate change, and in particular its link to extreme weather, has improved considerably, as has our picture of likely future changes. The need for deep and rapid cuts to emissions is even clearer than before. Based on the latest science, and taking into account Australia’s national circumstances, the Climate Council has concluded that Australia should reduce its emissions by 75% below 2005 levels by 2030, and achieve net zero emissions by 2035.”

## Discussion

Janet: The praxis of the Sundanese community resonates with the principles outlined in the ecocide law (Higgins et al. 2013) namely that multiple species need to be protected to live in harmony. The case study is discussed with reference to Ostrom’s 8 principles. In all respects the community seems to follow the 8 principles, but participation is carefully managed according to traditional rules which are applied to the management of rice growing. Innovation and participation are encouraged to the extent to which they are in line with their core values, namely achieving social and environmental harmony and balance. Economic principles of commodification are considered profane. The community of Ciptagelar can be characterised as being a participatory society with parallel roles for men and women embodied in the role of the chief as father and his wife as mother of the community who together take guidance from nature whom they worship. The rituals of reverence for the sacred goddess of rice show practical empirical elements as well as dance and song providing support for an oral culture and memory code that protects food security. This community demonstrates re-generative farming practices in harmony with the forest which they protect. High yields do not require the destruction of the forest, they require careful preparation of the soil and protection of robust seeds. The principles that this community follow are in line with the ideas that underpin the movement to support the introduction of an ecocide law (Higgins et al. 2013; McIntyre-Mills 2021a, b, c, d, e, f). The narrative to support the ecocide law is also supported in the companion paper on the work of the custodians in Venda (see McIntyre-Mills and Makaulule et al., 2022b; Lethole et al. forthcoming).

- **Principle 8 “Commons work best when nested within larger networks. Some things can be managed locally, but some might need wider regional co-operation …”**

From the discussion it appears that customs guide the organisation of society and that the community abides by wider Indonesian law to the extent that it does not conflict with their environmental values. It also seems that the Indonesian government could learn a great deal about zero emissions and re-generative living that goes beyond lip service to the United Nations Sustainable Development Goals.

In line with the approach demonstrated in Ciptagelar (and Venda) (McIntyre-Mills et al. 2022a, b, c, forthcoming), technology needs to serve the environment and people, thus values need to be at the centre of all governance and educational decisions. South Africa, Indonesia, and Australia would do well to be mindful of these lessons. In South Africa the deforestation that could accompany the implementation of so-called smart cities using digital technology and supported by, for example the Limpopo Provincial Government^26^ could lead to the loss of many ancient baobab trees on the border of Zimbabwe resulting in loss of habitat and loss of heritage. The tragic use of technology to destroy the environment is at odds with the potential to develop ways of sustaining the environment. The rolling black outs in South Africa cannot be solved by a reliance on a failing nuclear power station that is currently being de-commissioned^27^ as a Cape Talk back host in January speculated on ‘how safe it really is” and “ whether the managers can say anything other than that it is safe and well managed. “Would a manager of Fukushima, have said their plant was unsafe?” he asked and stressed that the managers were doing their best, but that the time had come to face the need to shift to renewable power supplies. Currently the licence to operate the plant should end soon, but the plant has been decommissioned to try to extend its operating life, but the process could have implications for safety and power supply. It would be much better to move towards a renewable supply of energy.

Unfortunately South Africa remains keen on old sources of energy, despite linking the sources with so-called ‘smart technology’. On the 25^th^ of February.^28^

“Limpopo province gave environmental authorisation to a China-backed proposal to spend more than $10 billion (R150 billion) building a 4 600 megawatt coal-fired power plant, a coking facility and ferroalloy and steel plants.”

The same article continues by explaining that the Musina-Makhado Special Economic Zone which borders Zimbabwe may not be permitted as the Chinese President Xi Jinping sated they would

“stop funding coal power abroad…”

It is hoped that this rational decision making will indeed prevail as a further report^29^ stresses that:

“Chinese President Xi Jinping said in a pre-recorded address at the United Nations General Assembly on Tuesday that China would help developing countries build green energy production and halt construction of coal power plants abroad.”

There is another way of doing things, which is documented and explored in several edited and sole authored Springer publications such as “Planetary Passport”, “Balancing Individualism and Collectivism”, “From Polarisation to Multispecies Relationships” and “ Democracy and Governance for Resourcing the Commons” and recently “Transformative Education.”

The need for participatory engagement in designs that support social and environmental justice are an obvious deficit of the planned cities^30^ that follow old style development and power over people and nature.

Technology ought to respond to designs that serve re-generation, hope and multispecies relationships. Where is the Afro-centrism in overseas projects that allow offshoring pollution and destruction?

Designs need to support the environment, not desecrate it. In Australia the young people who initially succeeded in taking the federal government to court for not showing a duty of care to protect young people, because the ministry of environment approved the introduction of more coal powered stations has since had their successful outcome overturned. The young people vow that they will continue to challenge the Australian federal government (Peel and Markey-Towler 2022). Examples such as the Ciptagelar community demonstrate that alternative ways of life to achieve zero emissions are sustainable and could be scaled up.

In this case study the community demonstrates how they understand that they are owned by the forest and the land and they do not commodify nature. They have a very different approach to agriculture and they demonstrate how to live in harmony with their environment.

**Fig. 11:**
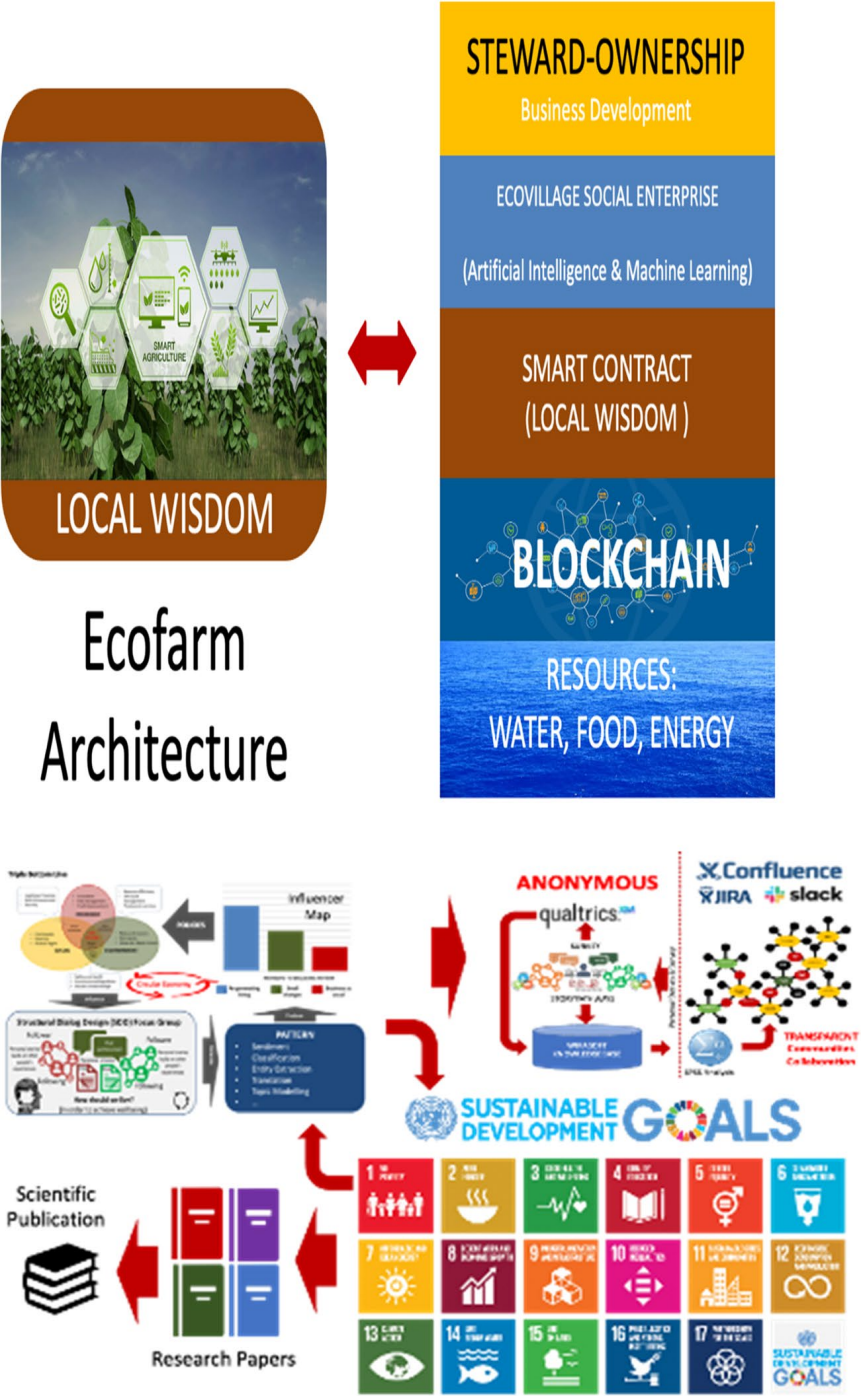
Stewardship agriculture, management of the commons and scaling up ways to manage the relationships across multiple species and find a way to balance individual and collective needs (Source Wirawan et al. 2022)

Their mode of living provides inspiration for other villages in Indonesia that could benefit from their lessons and which could be scaled up, in order to protect the natural environment in Indonesia. Wirawan, McIntyre-Mills et al are exploring the extent to which engagement to protect local communities based on local wisdom could be scaled up (Fig. 11):

## Conclusion

Janet: Engagement^31^ with Ciptagelar by Unpad staff and associates could be a step towards sharing local wisdom more widely. The time for learning with Indigenous leaders is overdue. The step could involve sharing lessons on the way the forest is being protected whilst also producing very successful rice crops (unlike other farmers using other techniques (even during times of drought) Furthermore, this case study could provide an example of low carbon living.

## Footnotes

1 https://www.onthecommons.org/magazine/elinor-ostroms-8-principles-managing-commmons/ https://earthbound.report/2018/01/15/elinor-ostroms-8-rules-for-managing-the-commons/

2 https://earthbound.report/2018/01/15/elinor-ostroms-8-rules-for-managing-the-commons/

3 Bisht, V. 294 subscribers https://www.youtube.com/watch?v=OEcMLEwaltc “This is an educational video about the commons scholarship pioneered by Elinor Ostrom, which provided a challenging alternative to Hardin's theory of the Tragedy of the Commons. "Commons" is a term used in resource economics to describe types of goods that can be accessed by anyone but their use by one party limits their availability to others. Examples of common-pool resources include pasture lands, freshwater, fisheries, forests, etc. Through her research with different communities across the world, Elinor Ostrom showed that it is possible for local communities to sustainably manage their common resources without any external intervention like state control or privatization.”

4 https://earthbound.report/2018/01/15/elinor-ostroms-8-rules-for-managing-the-commons/

5 https://www.youtube.com/watch?v=N8CXgBSQhcA

6 Elinor Ostrom on the Myth of Tragedy of the Commons https://www.youtube.com/watch?v=ybdvjvIH-1U

7 Recording Please find below the link to the recorded zoom session: https://drive.google.com/file/d/1NUkOsfWWkxo0YPH1btRUiApB2S679_lx/view?usp=sharing

8 (McIntyre-Mills 2019)

9 (McIntyre-Mills et al. 2022a; McIntyre-Mills 2019; McIntyre-Mills et al. 2019)

10 The *ngalalakon* ritual has distinct characteristics compared to regular migration. The community  migrates as a result of their traditional obligation, there are five aspects of *ngalalakon*, namely: the phase of *ngalalakon*, the characteristic of *ngalalakon*, the type of *ngalalakon*, the movement of *ngalalakon*, and the sign of *ngalalakon* (puteri & Kusdiwanggo 2018)*.* Oral history stated that the community started practice the nomadic tradition in 1368 from their first settlement in *Kasepuhan Cipatat Urug*, the latest movement was in 2000 to *Kasepuhan Ciptagelar.* The term *Ngalalakon* part of religious rituals, involving physical and cultural spaces. Interviews with the chief and village elders revealed that they have been moved for about 19 times under 11 different chiefs.

11 *Kasepuhan Ciptagelar* is formed based on the moving of the Chief (*Sesepuh girang)* from *Ciptarasa* village in Juli 2000. The name of Ciptagelar was taken from the chief’s name at that time, ‘Encup Sucipta”. ‘Cipta’ is the short name of the chief ‘Abah Anom’ (Sucipta), and ‘gelar’ means open or *pasrah.*

12 According to Astutik & Kusdiwanggo, 2019 who summarises the nomadic process or *ngalalakon* process as comprising the movement of buildings and traditional structures (1) rice barns (*Leuit Jimat) (2)* Community Buildings (*Bale warga) (3)* the paddy processing buildings (*Saung lisung) and (4)* the so-called ‘ big’ houses *(Imah gede).*

13 In our joint research on transformative education for re-generation (McIntyre-Mills and Corcoran Nantes, 2022) examples of education from Indonesia (see McIntyre-Mills, Wirawan and Widianingsih, 2022) and South Africa (see McIntyre-Mills, J.J. Lethole, P.V, Romm, NRA, Makaulule, M and Wirawan, R, 2021) are detailed in a paper called: 'Re-designing education for regeneration and wellbeing: Exploring the potential of digital engagement' demonstrate cyclical green economies.

14 Bisht, V. 294 subscribers https://www.youtube.com/watch?v=OEcMLEwaltc “This is an educational video about the commons scholarship pioneered by Elinor Ostrom, which provided a challenging alternative to Hardin's theory of the Tragedy of the Commons. "Commons" is a term used in resource economics to describe types of goods that can be accessed by anyone but their use by one party limits their availability to others. Examples of common-pool resources include pasture lands, freshwater, fisheries, forests, etc. Through her research with different communities across the world, Elinor Ostrom showed that it is possible for local communities to sustainably manage their common resources without any external intervention like state control or privatization.”

15 (Budiaman et al. 2021; Fadhilah et al. n.d.; Hermanto et al. 2012)

16 The community members spread around the Halimun Salak National Reserve, Cisolok Sub-District, Sukabumi Regency, West Java. Overall the area consists of 568 hamlets. Kasepuhan Ciptagelar village has distinctive characteristics, among others, in terms of location, settlement patterns, forms of architecture, as well as the traditions of the economic, social, and cultural communities still held fast by the community.

17 https://www.youtube.com/watch?v=ZV0NkADi2dc

18 https://www.google.com/search?q=abah+ugi+kasepuhan+ciptagelar&oq=abah+ugi&aqs=chrome.1.69i57j0i20i263i512l2j0i512j0i22i30l2j69i60l2.5743j0j7&sourceid=chrome&ie=UTF-8

19 https://earthbound.report/2018/01/15/elinor-ostroms-8-rules-for-managing-the-commons/

20 https://earthbound.report/2018/01/15/elinor-ostroms-8-rules-for-managing-the-commons/

21 https://earthbound.report/2018/01/15/elinor-ostroms-8-rules-for-managing-the-commons/

22 1.*Tanggal kerti kana beusi*, *tanggal kidang turun kujang*, which means that the community must prepare agricultural tools such as hoes, sickles, forks, and so on; 2. *Kidang ngrangsang ti wetan, kerti ngrangsang ti kulon* or *kidang-kerti paharep*-harep, a sign of a long summer, the right time to burn twigs and leaves in the farm 3. *Kerti mudun matang mencrang*, the time to plant rice 4. *Kidang and kerti ka kulon*, which means the rainy season will soon arrive. 5. *Kidang medang turun kukang*, means that the time when pests and other plant diseases will attack rice plants.

23 https://earthbound.report/2018/01/15/elinor-ostroms-8-rules-for-managing-the-commons/

24 https://www.climatecouncil.org.au/resources/ipcc-sixth-assessment-report-explained/?atb=DSA01c&gclid=Cj0KCQjwz7uRBhDRARIsAFqjulkHXfoVyCQrgnFbo8QE0mIT-wtAx6b1hBivnERaWPPxXsfP2cwdiw0aAoPUEALw_wcB

25 https://www.climatecouncil.org.au/resources/ipcc-sixth-assessment-report-explained/?atb=DSA01c&gclid=Cj0KCQjwz7uRBhDRARIsAFqjulkHXfoVyCQrgnFbo8QE0mIT-wtAx6b1hBivnERaWPPxXsfP2cwdiw0aAoPUEALw_wcB

26 https://www.news24.com/fin24/economy/eskom/limpopo-clears-way-for-r150bn-chinese-coal-complex-20220225

27 https://www.news24.com/fin24/economy/eskom/eskom-warns-power-system-may-come-under-more-strain-as-koeberg-units-go-offline-20220115 https://www.dailymaverick.co.za/article/2022-01-09-nuclear-power-station-life-extension-project-running-late-even-before-it-starts/

28 https://www.news24.com/fin24/economy/eskom/limpopo-clears-way-for-r150bn-chinese-coal-complex-20220225

29 https://www.reuters.com/business/energy/chinas-overseas-coal-power-retreat-could-wipe-out-50-bln-investment-2021-09-22/

30 The concepts of “smart cities’, “ smart governance” in South Africa range from small shuttle bus services on the University of Cape Town’s campus to attempts to manage wider infrastructure projects in Cape Town to managing aspects of the Covid vaccine rollout and also include fully planned cities such as Water Fall in Gauteng as well as the proposed city in Limpopo on the border of Zimbabwe.

31 We acknowledge the support of Prof Widya Sumandinata, Dean of the Faculty of Social and Political Sciences, Universitas Padjadjaran and International Relations Department who helped to make the research possible through supporting community outreach with Abah Ugi, the Chief of Kasepuhan Chiptagelar who has worked for many years with Gustaff Iskander who in turn helped to introduce the first and second authors to the community as well as Wirawan who ( through engaging with us on ways the low carbon living (exemplified in this case study) could be applied more widely as suggested in the conceptual diagrams provided by Wirawan.
